# Human α3β4 Neuronal Nicotinic Receptors Show Different Stoichiometry if They Are Expressed in *Xenopus* Oocytes or Mammalian HEK293 Cells

**DOI:** 10.1371/journal.pone.0013611

**Published:** 2010-10-26

**Authors:** Paraskevi Krashia, Mirko Moroni, Steven Broadbent, Giovanna Hofmann, Sebastian Kracun, Marco Beato, Paul J. Groot-Kormelink, Lucia G. Sivilotti

**Affiliations:** 1 Department of Neuroscience, Physiology and Pharmacology, University College London, London, United Kingdom; 2 Institute for Cell and Molecular Biosciences, The Medical School, Newcastle University, Newcastle upon Tyne, United Kingdom; 3 Department of Physiology, University of California Los Angeles, Los Angeles, California, United States of America; 4 Novartis Horsham Research Centre, Novartis Institutes for Biomedical Research, Horsham, United Kingdom; Vrije Universiteit Amsterdam, Netherlands

## Abstract

**Background:**

The neuronal nicotinic receptors that mediate excitatory transmission in autonomic ganglia are thought to be formed mainly by the α3 and β4 subunits. Expressing this composition in oocytes fails to reproduce the properties of ganglionic receptors, which may also incorporate the α5 and/or β2 subunits. We compared the properties of human α3β4 neuronal nicotinic receptors expressed in Human embryonic kidney cells (HEK293) and in *Xenopus* oocytes, to examine the effect of the expression system and α∶β subunit ratio.

**Methodology/Principal Findings:**

Two distinct channel forms were observed: these are likely to correspond to different stoichiometries of the receptor, with two or three copies of the α subunit, as reported for α4β2 channels. This interpretation is supported by the pattern of change in acetylcholine (ACh) sensitivity observed when a hydrophilic Leu to Thr mutation was inserted in position 9′ of the second transmembrane domain, as the effect of mutating the more abundant subunit is greater. Unlike α4β2 channels, for α3β4 receptors the putative two-α form is the predominant one in oocytes (at 1∶1 α∶β cRNA ratio). This two-α form has a slightly higher ACh sensitivity (about 3-fold in oocytes), and displays potentiation by zinc. The putative three-α form is the predominant one in HEK cells transfected with a 1∶1 α∶β DNA ratio or in oocytes at 9∶1 α∶β RNA ratio, and is more sensitive to dimethylphenylpiperazinium (DMPP) than to ACh. In outside-out single-channel recordings, the putative two-α form opened to distinctive long bursts (100 ms or more) with low conductance (26 pS), whereas the three-α form gave rise to short bursts (14 ms) of high conductance (39 pS).

**Conclusions/Significance:**

Like other neuronal nicotinic receptors, the α3β4 receptor can exist in two different stoichiometries, depending on whether it is expressed in oocytes or in mammalian cell lines and on the ratio of subunits transfected.

## Introduction

Heterologous expression of muscle nicotinic receptors produces recombinant channels whose properties are similar to those of native channels from the neuromuscular junction. These properties are broadly similar for channels expressed in the two main heterologous expression systems, *Xenopus* oocytes and mammalian cell lines. The situation is different for the other main form of nicotinic receptor at peripheral synapses, the ganglion receptor. The main subunits contributing to this channel are α3 and β4 (probably together with α5 and β2 [1]–[3]). Receptors expressed in oocytes from these subunits resemble those from native autonomic ganglia in macroscopic pharmacology [4], but are very different at single-channel level. Most oocyte-expressed α3β4 channels open to a smaller conductance and produce much longer bursts than native receptors [5]. When the α3 and β4 subunits were stably transfected in mouse fibroblasts at a 1∶1 ratio, our outside-out recordings showed both oocyte-like channels and an additional, ganglion-like class of openings [6]. At the time, we did not have an explanation for this phenomenon, as obtaining transient expression of neuronal nicotinic subunits at sufficient levels was problematic.

The central-type α4β2 neuronal nicotinic combination can exist in two stoichiometries, which contain either two or three copies of the α4 subunit in the channel pentamer [7], as first suggested by Papke and co-workers for α2β2 [8]. The two forms of the α4β2 receptor can be distinguished in that the two-α form is activated at much lower ACh concentrations, has a different sensitivity to other agonists and does not display enhancement of submaximal ACh responses in the presence of low Zn^2+^ concentrations [9], [10]. Given that in the Cys-loop superfamily binding sites are at the interface of subunits, different subunit stoichiometries result in pharmacological differences in binding sites (see for GABA_A_ receptors [11]).

Earlier work on oocytes injected with α3 and β4 subunit cRNA has indicated the presence of a heterogeneous channel population [12]. Here we present evidence that α3β4 receptors, like the α4β2 receptor, can also exist in two different stoichiometries, whose different properties explain our old results [5], [6]. This is shown by data from receptors containing 9′ reporter mutations in either the α or β subunit in both oocytes and transiently-transfected HEK293 cells. We also provide a characterisation of the two forms of the α3β4 receptor, exploiting the fact that the stoichiometry of oocyte-expressed receptors can be manipulated by changing α∶β cRNA ratios. When injections are carried out with equal amounts of α3 and β4 cRNA, the putative two-α form of the channel predominates in oocytes [13], whereas the three-α form is more abundant in HEK cells. The most distinctive macroscopic property of the two-α form is that it displays both potentiation and inhibition in response to Zn^2+^ (only inhibition is observed in the three-α form). In addition to that, single-channel recordings from HEK cells indicate that the “oocyte-like” long-burst, low-conductance channel corresponds to the two-α form, whereas the short-burst, high conductance class (“ganglion-like”, see [5], [6]) reflects the activity of pentamers containing three α subunits.

## Results

### Human α3β4 nAChR expressed in HEK293 cells contain three α subunits

**Figure 1:**
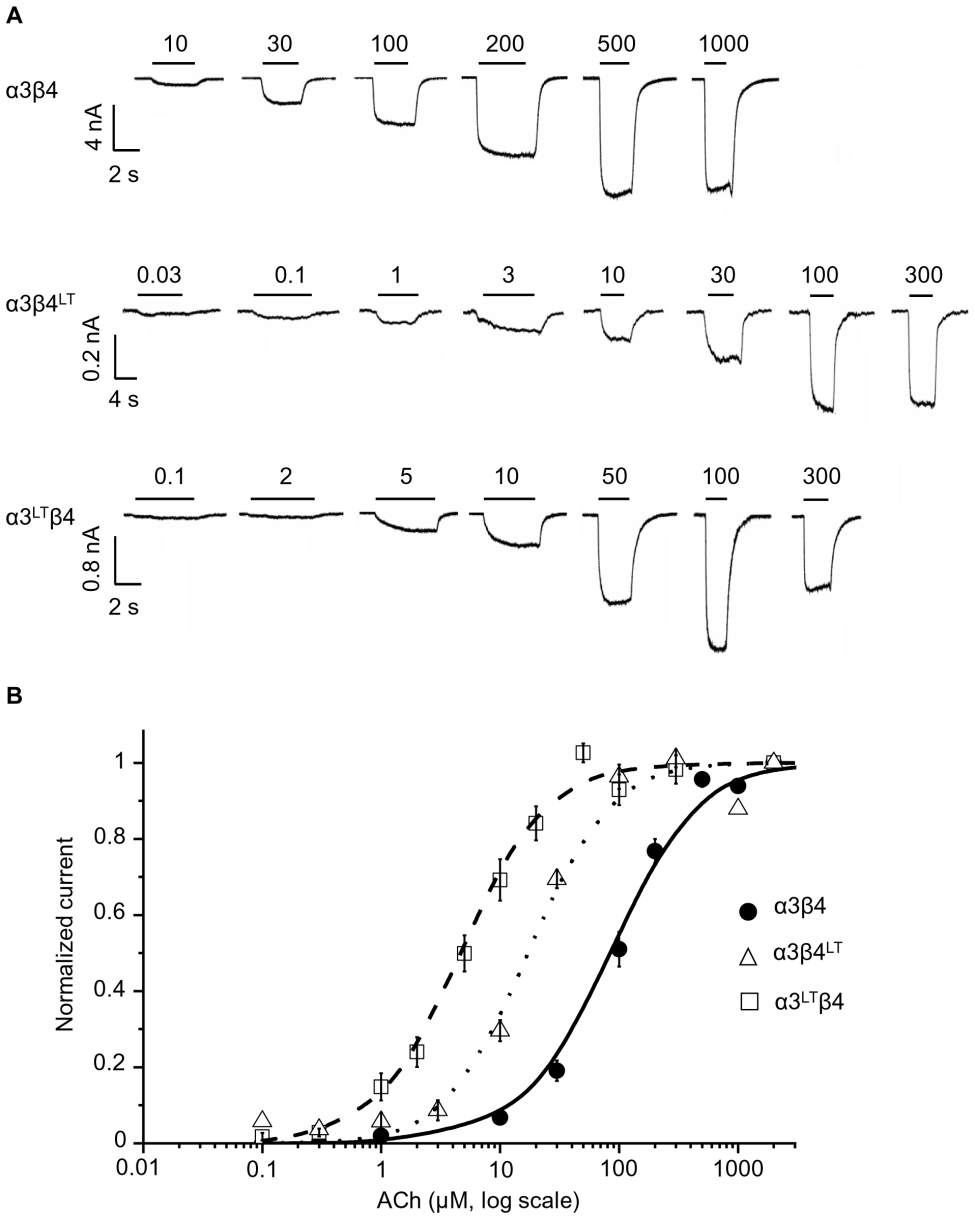
In HEK-expressed α3β4 receptors, the 9′LT mutation has a greater effect if inserted in α3. Traces in (A) are whole cell inward currents elicited by different ACh concentrations applied to cells expressing wild type (top), α3β4^LT^ (middle) or α3^LT^β4 (bottom) channels. The bars above the traces show the duration of the agonist applications and the agonist concentration (in µM). Cells were transfected with an α3 to β4 ratio of 1∶1 and held at −30 mV. Dose-response curves are shown in (B) for each receptor combination. The data were averaged after normalisation to the fitted maximum for each experiment. The lines show Hill equation fits (see Methods) to the pooled normalised data (*n* = 6–8).

HEK293 cells were transfected with equal amounts of the α3 and β4 subunit cDNA to express α3β4 receptors. We characterised the change in ACh sensitivity caused by introducing a reporter mutation in either the α or the β subunit (see Figure 1). This mutation (9′ LT) replaces the conserved leucine in the middle of the pore-lining second transmembrane domain (M2) with a hydrophilic threonine and increases agonist potency in a manner proportional to the number of mutations in the channel [13], [14]. The dose-response curves in Figure 1B (obtained from the peak inward currents elicited by ACh, see the traces in panel A) show that the greater shift in ACh potency is seen when the mutation is in the α subunit. Thus, α3^LT^β4 receptors are almost 20 times more sensitive to ACh than wild-type channels, with an *EC*_50_ of 5.5±0.9 vs. 91.1±10.7 µM, respectively (*n* = 7 and 8, respectively, see Table 1). Mutating the β subunit had a significantly smaller effect, reducing *EC*_50_ by about 5-fold, to 18.6±1.9 µM for α3β4^LT^ receptors (*n* = 6; significance from the confidence intervals for the potency ratios, Table 1). This strongly suggests that HEK-expressed α3β4 receptors contain more α than β subunits, in contrast with our observations in oocyte-expressed α3β4 receptors, where the same approach indicated a 2α∶3β stoichiometry [13].

**Table 1:** Effects of 9′ LT mutation on the ACh dose-response curve of human α3β4 receptors expressed in HEK293 cells.

Subtype	*EC*_50_ (µM)	*I_max_* (nA)	Hill slope	Potency ratio	Square root of potency ratio	Cube root of potency ratio	*n*
α3β4	91.1±10.7	6.67±1.63	1.65±0.23	1	-	-	8
α3β4^LT^	18.6±1.93	0.48±0.14	1.43±0.11	5.13 [4.31–6.08]	2.26 [2.08–2.47]	-	6
α3^LT^β4	5.51±0.91	2.21±0.77	1.27±0.04	18.6 [15.1–22.9]	-	2.65 [2.47–2.84]	7

*EC*_50_, *I_max_* and Hill slope are means ± standard error of the mean of separate fits of each dose-response curve to the Hill equation. Potency ratios relative to the wild type curve were estimated from the parallel fits in which the curves were constrained to have equal Hill slopes. The 2-unit likelihood intervals for the ratios are shown in square brackets.

### Injection of oocytes with different α∶β cRNA ratios produces receptors with different stoichiometry

In our previous work in oocytes, cells were injected with equal amounts of α3 and β4 subunit cRNA [4], [5], [13]. More recent studies proved that changing the expressed ratio of α∶β changes the subunit stoichiometry of α4β2 nicotinic receptors in oocytes [9], [15] and we hypothesised that this may occur also for α3β4 receptors.

**Figure 2:**
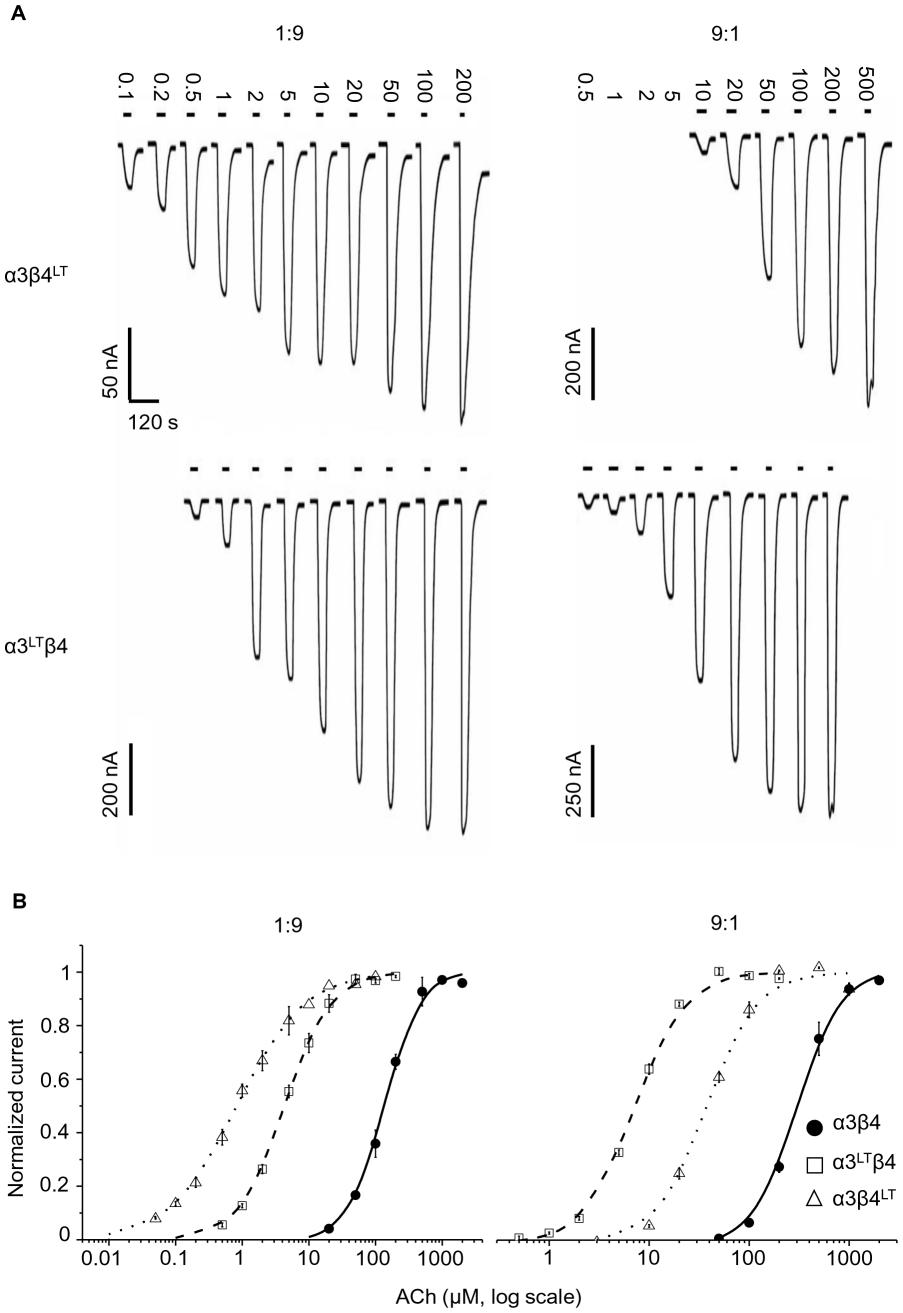
The effect of the 9′ LT mutation in oocytes depends on the α3∶β4 subunit ratio. Traces in (A) represent inward currents elicited by different concentrations of ACh (in µM) bath applied (duration shown as a solid bar). Cells were held at −70 mV. Pooled normalised dose-response curves, fitted with the Hill equation with free parameters are shown in (B) for oocytes injected with 1∶9 (left) or 9∶1 (right) α3 to β4 cRNA ratio. The 1∶9 subunit ratio produces wild-type receptors that are more sensitive to ACh. The effect of the mutation is greater when it is carried by the β4 subunit if β4 is overexpressed (1∶9) and when the α3 subunit is mutated if the α3 subunit is overexpressed (9∶1).

Figure 2 shows inward currents elicited by ACh in oocytes expressing α3^LT^β4 or α3β4^LT^ receptors at α∶β cRNA injection ratios of 1∶9 and 9∶1 and the respective dose-response curves. When a subunit ratio of 1α∶9β was used (left side of Figure 2), mean *EC*_50_ values were very similar to those observed when equal amounts of α and β cRNA were injected (Table 2 and [13]). In particular, the greater increase in agonist sensitivity, approximately 150-fold, occurred when the reporter mutation was inserted in the β subunit (top half of Table 2). The effect of the mutation on the ACh dose-response curve was very different when the α subunit cRNA was injected in a 9-fold excess to the β subunit (right side of Figure 2). This time the pattern was similar to that seen for receptors expressed in mammalian cells, as the greater increase in ACh sensitivity was seen for α3^LT^β4 receptors, with a leftward shift of 47-fold (bottom of Table 2), significantly greater than the 10-fold shift produced by inserting the mutation in the β subunit (cf. the confidence intervals shown in Table 2). This is what would be expected if excess α cRNA forces the oocyte to produce receptors with a three-α and two-β stoichiometry, and overrides the oocyte tendency to express receptors with two α and three β in the pentamer.

**Table 2:** Effects of the 9′ LT mutation on the ACh dose-response curve of human α3β4 receptors expressed in oocytes at extreme ratios.

α∶β ratio	Subtype	*EC*_50_ (µM)	*I_max_* (nA)	Hill slope	Potency ratio	Square root of potency ratio	Cube root of potency ratio	*n*
**1∶1**	α3β4	172±8.4	2050±301	1.56±0.07	1	-	-	5
**1∶9**	α3β4	138±13.7	1270±316	1.79±0.14	1	-	-	4
	α3^LT^β4	4.48±0.38	1060±238	1.33±0.09	36.8 [33.8–40.0]	6.1 [5.8–6.3]	-	5
	α3β4^LT^	0.92±0.13	226±54	0.90±0.12	-	-		4
**9∶1**	α3β4	309±27.8	686±213	2.41±0.21	1	-	-	4
	α3β4^LT^	37.7±1.70	720±264	1.91±0.10	9.6 [8.88–10.3]	3.1 [2.98–3.21]	-	3
	α3^LT^β4	7.21±0.21	1580±407	1.91±0.05	46.80 [43.7–50.1]	-	3.6 [3.52–3.69]	5

*EC*_50_, *I_max_* and Hill slope are means ± standard error of the mean of separate fits of each dose-response curve to the Hill equation. Potency ratios are expressed relative to the wild-type curve and were estimated from parallel fits to the Hill equation (note that the dose-response curve for the 1∶9 α3β4^LT^ receptor was too shallow to allow parallel fits do be done without too much distortion, see also [13]. The 2-unit likelihood intervals for the potency ratios are shown in brackets.

**Figure 3:**
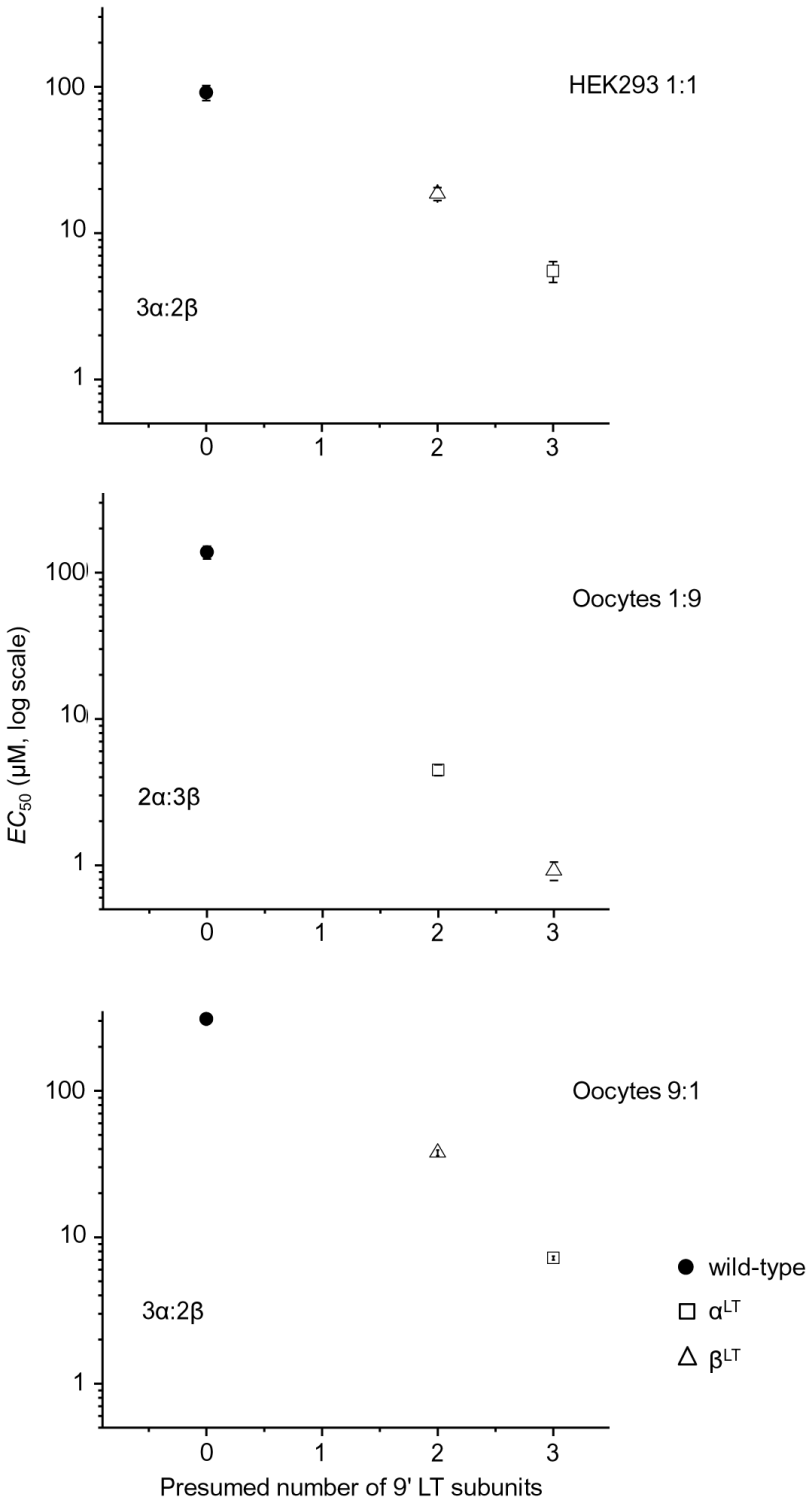
Relationship between the average *EC*_50_ values and the presumed number of 9′ LT mutations. Each point is plotted according to the stoichiometry we suggest for each cell type and subunit ratio. The assumed stoichiometry is stated on the plot. The linear relationship between the *EC*_50_ values and the number of mutated subunits confirms that the stoichiometry we assume is plausible.

In the simplest case of a symmetrical channel made of equivalent subunits, each mutation copy would produce an equal “unitary” change in the free energy of gating, and hence a *r*-fold “unitary” change in *EC*_50_ values. Two or three copies of the mutation would shift *EC*_50_ by *r* ^2^ or *r* ^3^-fold, respectively. This gives rise to a linear relation between the logarithm of the *EC*_50_ values and the number of mutant subunits incorporated in the pentamer. Figure 3 shows that this relation holds for HEK- and oocyte-expressed receptors with up to three copies of the mutation. These data are consistent with our interpretation that α3β4 receptors from HEK and from oocytes injected with the 9∶1 α∶β ratio contain three copies of the α subunit, whereas receptors from oocytes injected with a 1∶1 or a 1∶9 ratio contain only two copies of the α subunit. Note also that our estimates of the elementary change in *EC*_50_, *r* (from the square or cube root of the potency ratios), are similar for putative three-α receptors whether they are expressed in HEK or in oocytes (see Tables 1 and 2).

Our results on α3β4 receptors, therefore, agree with those on α4β2 receptors [9], [15]: depending on the α3 to β4 cRNA ratio used, oocytes can express receptors that contain either two or three copies of the α subunit in the pentamer.

### Receptors expressed in oocytes with a ratio of 1∶9 and 9∶1 have different sensitivity to DMPP

The *EC*_50_ values in Table 2 also show that wild-type α3β4 receptors expressed with extreme ratios have different ACh sensitivity, 137.8±13.7 vs. 309.5±27.8 µM (*n* = 4 for both, *p* = 0.0015, two-tailed Student's *t* test) for the 1∶9 and 9∶1 ratios, respectively. In α4β2 receptors this difference is much greater (80-fold or more) but in the same direction [7], [9]. It is possible that the two-α and the three-α form of α3β4 differ in other properties, some of which may be useful as tools in characterising native receptors. We therefore characterised the sensitivity of the two forms to a range of nicotinic agonists.

**Figure 4:**
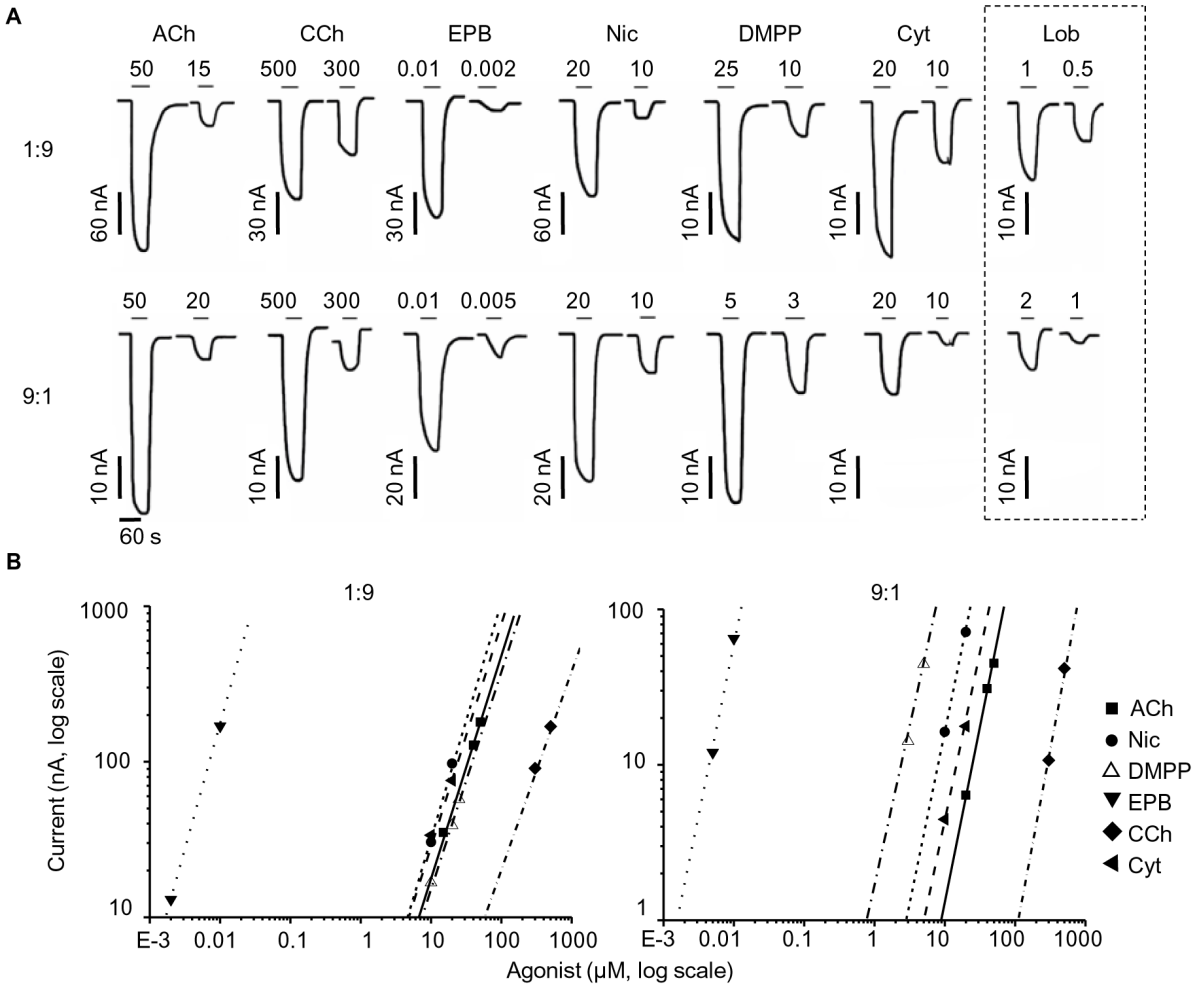
The two different receptor stoichiometries expressed in oocytes differ in their DMPP sensitivity. The traces in (A) are examples of inward currents elicited by low agonist concentrations (in µM) and recorded from oocytes injected with a subunit ratio of 1∶9 (top traces) and 9∶1 (bottom). The duration of each agonist application is shown above each trace (solid bar). All agonists (CCh, carbachol; EPB epibatidine; Nic, nicotine; DMPP, dimethylphenylpiperazinium; Cyt, cytisine; LOB, lobeline) were tested on the same oocyte (held at −70 mV), with the exception of lobeline (shown on the right, recorded from a different cell but with similar initial ACh current as the cell on the left, for both subunit ratios). The log-log plots in (B) are partial dose-response curves for the experiments shown in A (left). Note the increase in the potency of DMPP (10-fold leftward shift of the dose-response curve) in the 9∶1 ratio.

Figure 4A shows examples of inward currents elicited by low concentrations of nicotinic agonists in oocytes injected with a ratio of 1∶9 (first row) or 9∶1 α∶β subunit cRNA (second row). Panel B shows partial dose-response curves as log-log plots (effectively the foot of the dose-response curve) to six agonists obtained in two representative experiments (corresponding to the traces in A, except for the lobeline responses that are from two different experiments, see legend). If we refer to the standard agonist, ACh (continuous lines in Fig. 4B), there are two agonists that stand out, epibatidine and carbachol. On both types of receptors, epibatidine is very much more potent than ACh (by more than 6000-fold), whereas carbachol is much less potent (by about 10-fold, see Table 3). The other agonists tested in these experiments, nicotine, DMPP and cytisine, are very much closer to ACh in their potency. Remarkably, the rank order of potency for the agonists we tested is very similar for the putative two-α and the three-α receptors, epibatidine≫lobeline>nicotine∼cytisine>ACh>carbachol, in descending order of potency, with the single exception of DMPP (see Table 3). DMPP and ACh were equipotent (potency ratio 0.99±0.023) in 1∶9 oocytes (*n* = 9), but DMPP was ∼10 times more potent than ACh (10±0.62, *n* = 8) when cells were injected in a 9∶1 ratio. The rank order of potency for the 1∶9 receptors is almost identical to the one we reported for 1∶1 receptors [13], in agreement with our conclusion from the reporter mutation experiments that in oocytes both the 1∶1 and the 1∶9 ratio of subunits give rise to receptors with two copies of α in the pentamer.

**Table 3:** Potency ratio values for a range of nicotinic agonists on human α3β4 neuronal nicotinic receptors expressed in oocytes.

α∶β ratio	Agonist	Potency ratio	95% confidence intervals	Log-log slope	*n*
**1∶9**	Epibatidine	6290±815	[4820–9060]	1.35±0.05	8
	Lobeline	7.35±0.56	[6.20–9.03]	1.06±0.09	8
	Nicotine	1.43±0.14	[1.16–1.86]	1.43±0.06	8
	Cytisine	1.40±0.15	[1.13–1.82]	1.07±0.04	10
	ACh	1	-	1.45±0.07	22
	DMPP	0.99±0.02	[0.95–1.05]	1.21±0.08	9
	Carbachol	0.094±0.003	[0.088–0.100]	1.07±0.09	12
**9∶1**	Epibatidine	6260±573	[5110–8070]	2.04±0.17	7
	Lobeline	17.7±2.22	[13.4–26.1]	2.04±0.17	6
	Nicotine	2.95±0.14	[2.65–3.33]	2.33±0.11	9
	Cytisine	1.92±0.16	[1.58–2.46]	1.77±0.11	6
	ACh	1	-	2.19±0.13	19
	DMPP	10.2±0.62	[8.91–11.9]	2.31±0.10	8
	Carbachol	0.093±0.004	[0.083–0.102]	2.37±0.15	6

Numbers shown are average values ± standard error of the mean. Potency ratios, relative to the standard agonist ACh, express how much more potent than ACh an agonist is at a comparable level of response. The 2-unit likelihood intervals for the potency ratios are shown in brackets.

### In oocytes, Zn^2+^potentiation of α3β4 nicotinic currents is not seen when the α subunit is overexpressed

In the α4β2 receptor, Zn^2+^ potentiation of submaximal ACh responses is stoichiometry-specific and occurs only in receptors with three copies of the α subunit [10]. Specific amino acids on the α4-α4 interface are necessary for potentiation, in particular a glutamate residue, in loop C, on the + side of the subunit-subunit interface, together with a histidine residue, in loop F, on the − side. By analogy with the α4β2 combination, we hypothesised that the potentiating effect of Zn^2+^ might be stoichiometry-specific also in the α3β4 receptor. We verified this hypothesis by testing the effects of Zn^2+^ on the two forms of α3β4 expressed in oocytes with 9∶1 or 1∶9 α∶β cRNA ratios.

**Figure 5:**
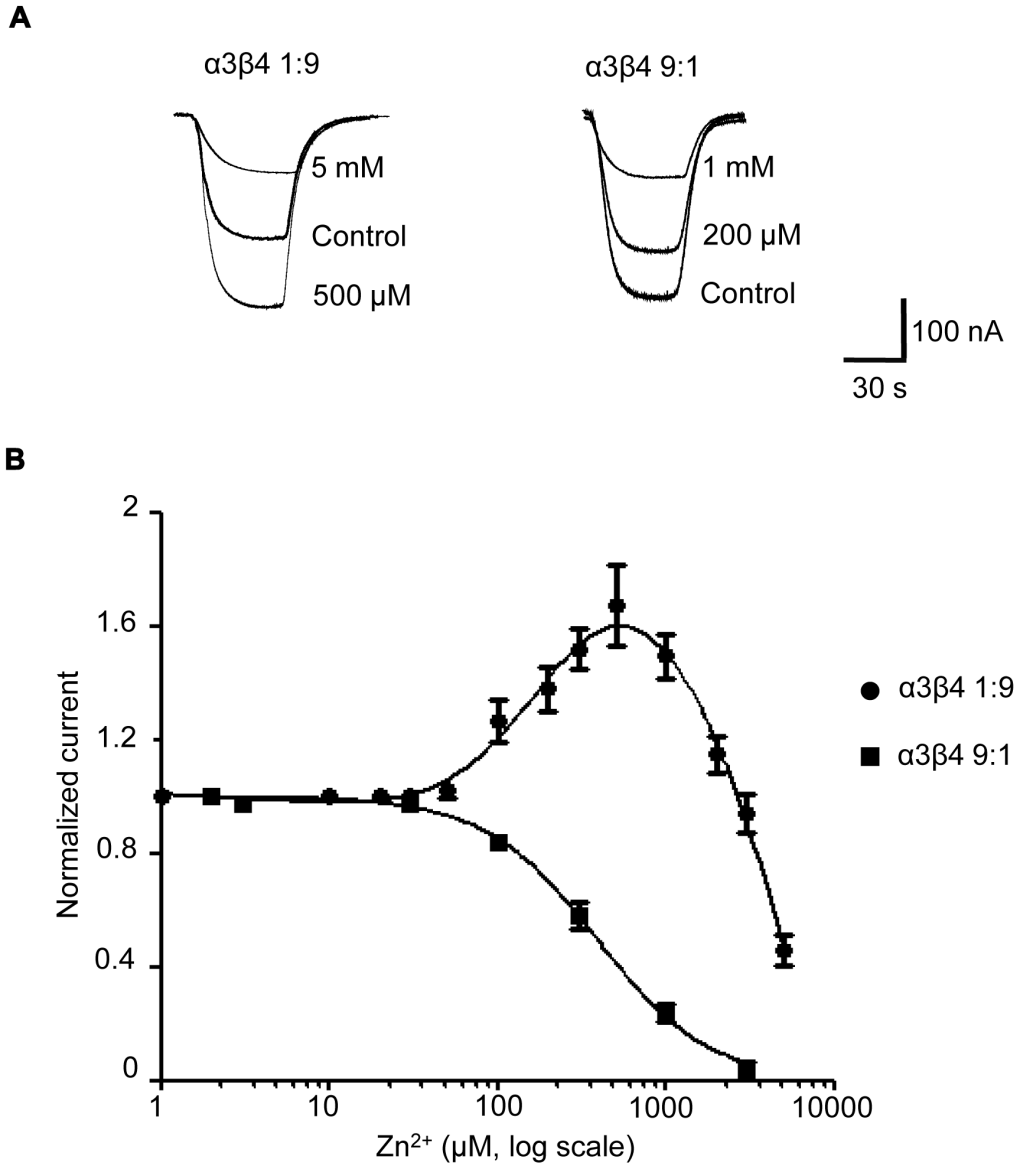
The two-α and three-α form of the α3β4 receptor differ in their response to Zn^2+^ modulation. Traces in (A) are typical currents activated by an *EC*_20_ concentration of ACh on the two-α receptor (left, 1∶9 α∶β ratio) and the three-α receptor (right, 9∶1 ratio) in the presence of increasing concentrations of Zn^2+^. Zn^2+^ was pre-applied to oocytes for 30 s before it was applied together with ACh. (B) Zn^2+^ concentration-response curves for the three-α receptor (filled squares) and the two-α receptor (filled circles). Curves from different cells were pooled and fitted to the Hill equation and to the sum of two Hill equations, respectively. Note that Zn^2+^ potentiated only the ACh responses of oocytes expressing the two-α stoichiometry.

The traces in Figure 5A show the effect of increasing concentrations of Zn^2+^ on the inward currents elicited by applying ACh at *EC*_20_ to receptors with different stoichiometries. Normalised average responses are also plotted as dose-response curves in panel B of the Figure. As we hypothesized, no Zn^2+^ enhancement was seen for the putative three-α form of the α3β4 receptor (α3β4 9∶1, filled squares in Fig. 5B): Zn^2+^ only inhibited ACh currents with an *IC*_50_ of 400±50 µM and an (absolute) Hill slope value of 1.4±0.1 (*n* = 4). In contrast to that, Zn^2+^ had a complex effect on the putative two-α form of the α3β4 receptor (α3β4 1∶9, filled circles in Fig. 5B). Lower Zn^2+^ concentrations (0.5 to 2 mM) enhanced ACh responses by up to 70±13% with *EC*_50_ and Hill slope values of 168.2±14 µM and 1.8±0.2, respectively (*n* = 5). Concentrations higher than 2 mM inhibited ACh currents with *IC*_50_ and (absolute) Hill slope value for the inhibition of 3.2±0.3 mM and 2.1±0.2, respectively.

Our observation that ACh responses from the putative two-α form of α3β4 receptors can be either enhanced or inhibited, depending on the Zn^2+^ concentration, agree with similar results in oocytes injected with α3 and β4 cRNA in a 1∶1 ratio [16]. This is consistent with our finding that oocytes injected with equal amount of α3 and β4 cRNA express preferentially the two-α form of the receptor [13].

### Receptors expressed in HEK293 cells with a ratio of 1∶9 and 9∶1 differ in their single-channel properties

**Figure 6:**
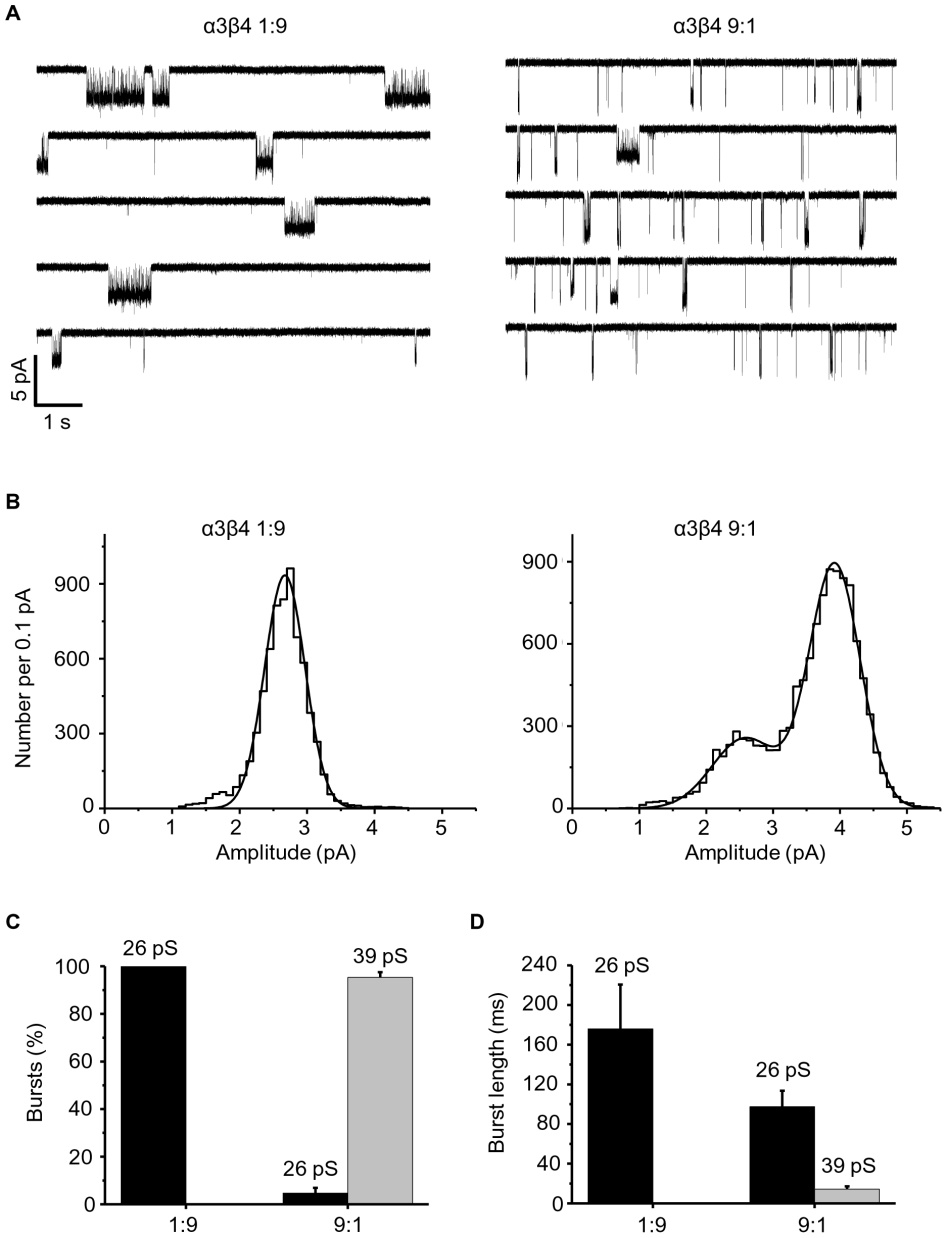
Single-channel properties of α3β4 receptors expressed from extreme ratios in HEK293 cells. (A) Examples of outside-out currents elicited by 5 µM ACh, from cells transfected with an α3 to β4 cDNA ratio of 1∶9 (left) and 9∶1 (right). Patches were held at −100 mV. The histograms of fitted amplitudes corresponding to these recordings are shown in (B). These are fitted with Gaussian curves to give the peak current amplitudes and the areas under each curve (for these two patches the values are 2.7±0.3 pA, area 100% for 1∶9; 2.6±0.5 pA, area 27% and 3.9±0.4 pA, area 73% for 9∶1). The histogram on the left (C) shows the proportion of bursts (as % of the total number of bursts from all experiments, pooled) at each chord conductance for the two subunit ratios. The histogram on the right (D) shows the difference in the duration of the bursts to different conductances for the two ratios.

Oocyte-expressed α3β4 channels open mostly in long bursts to a low (22 pS) conductance [5]: the results shown above suggest that these openings reflect the activity of a two-α form of the receptor (see also ref. [12]). This sort of channel is common also in stably-transfected mammalian fibroblasts, where we observed an additional type of channel activity, with shorter bursts and a higher conductance of 36 pS [6]. Our new data on HEK293 cells show that they preferentially express another form of the receptor, and raise the possibility that the short-burst, high conductance channels correspond to a three-α stoichiometry. This hypothesis is confirmed by the data in Figure 6.

The traces in Figure 6A are representative single-channel currents evoked by the application of 5 µM ACh to outside-out patches at −100 mV. HEK cells were transfected with α3 and β4 cDNA at a ratio of 1∶9 (left) or 9∶1 (right). Openings in 1∶9 patches are smaller in amplitude and occur in longer bursts, resembling the channels we characterised in oocytes, i.e. the putative two-α form of the channel. On the other hand, for 9∶1 transfected cells, two different classes of openings occur: one is similar in amplitude and burst length to the putative two-α form observed in the 1∶9 cells, whereas a second class opens in shorter bursts to a bigger conductance. These two classes of openings are never linked to each other by direct transitions (data not shown), and therefore they are likely to be produced by different receptor molecules.

The data from the patches in Figure 6A are plotted as fitted amplitude histograms in Figure 6B, where each opening is counted as one data point. The continuous curves are the fits with one (1∶9) or two (9∶1) Gaussian components. All patches from the 1∶9 transfection had one component with average amplitude of 2.6±0.1 pA (corresponding to a chord conductance of 26 pS; *n* = 7). Low conductance openings were also present in three out of five 9∶1 patches, where their average amplitude was 2.6±0.1 pA (area 24±6%), but even when present were less common than high conductance openings, which had an amplitude of 3.9±0.04 pA (39 pS; four out of five patches) and an area of 78±8%.

Because the amplitude histograms in panel B are based on openings, they emphasize the contribution of channels that open in prolonged bursts of many openings, such as the low conductance, putative two-α receptors. It is informative to re-plot these data counting the number of *bursts* for each conductance (of course, neither method allows us to estimate the number of channels in each subtype). Figure 6C shows that in the three 9∶1 patches that did have low conductance openings, only a small proportion of bursts, 7.8±1.8% (*n* = 3), belong to low conductance channels. Figure 6D quantifies another property of these two classes of channels, namely that the smaller openings always occur in distinctive long bursts (176±45 ms and 98±16 ms for the 1∶9 and 9∶1 transfections, *n* = 7 and 3 patches, respectively). The higher conductance of 39 pS, which predominates in 9∶1 cells, has a shorter burst length of 14±2 ms.

## Discussion

Depending on expression system and α∶β subunit ratios, recombinant α3β4 neuronal nicotinic receptors contain either two or three copies of the α subunit. This is similar to α4β2 receptors, except that α3β4 channels favour the three-α form in HEK cells and the two-α form in oocytes. The two forms are similar in agonist sensitivity and agonist potency, but only the putative two-α form is susceptible to enhancement by low zinc concentrations. Furthermore, the two stoichiometries are markedly different in both single-channel conductance and kinetics.

### The two forms of the α3β4 receptor have different α∶β stoichiometry

Early suggestions that neuronal nicotinic receptors exist in different stoichiometries [8], [15] were proven for α4β2 receptors [7], where the two forms differ by up to 100-fold in ACh sensitivity [9], [17], [18].

Heterogeneity in the properties of α3β4 receptors across expression systems may also be explained by different stoichiometries [6]. This is supported by several lines of evidence. Firstly, the effects of the 9′ LT mutation are different in the two expression systems and in oocytes they depend on α∶β cRNA ratios. In the past we showed by this approach that a single copy of β3 is incorporated in α3β4β3 nicotinic receptors [13], a finding recently confirmed by detecting FRET between subunits bearing appropriate fluorescent probes [19]. In the present experiments, *EC*_50_ shifts were consistent, with a roughly linear relation between log *EC*_50_ and number of mutation copies (Figure 3). A second line of evidence comes from the effects of zinc, which enhances only the putative two-α form. In α4β2 receptors, Zn^2+^ potentiation requires an α4(+)/α4(−) interface, present only in the three-α form. The key two residues on the opposite sides of the interface are α4 His165 (Loop F, − side) and α4 Glu194 (Loop C, + side) [10]. The question is which subunit interface mediates zinc enhancement in the α3β4 receptor. It cannot be the β4(+)/α3(−) interface (present in both stoichiometries), but it cannot be the α3(+)/α3(−) interface either (present only in the three-α form), as it lacks the key residues identified in α4. By exclusion, it would seem that the β4(+)/β4(−) interface could mediate Zn^2+^ potentiation, as it is present only in the two-α stoichiometry. This hypothesis could be tested by site-directed mutagenesis.

### Macroscopic properties of the two forms of the α3β4 receptor

α4β2 receptors from 1∶1 α∶β transfections are a mixture of stoichiometries, but three-α, low-sensitivity channels predominate [7], [9], [18]. Contrary to that, for α3β4 channels from 1∶1 α∶β ratios, the putative two-α form is the predominant one in oocytes and the three-α in HEK cells. The different behaviour could be due to differences in the binding of α3 and α4 to chaperone proteins (such as 14-3-3, known to affect the proportion of the two forms of α4β2 [20], given that the α-subunit intracellular domain is not conserved.

The two forms of the α4β2 receptor differ greatly in pharmacology, cation permeability and response to chronic nicotine [7], [9], [21], [22]. Remarkably, this is not the case for α3β4 receptors, at least at macroscopic level. In oocytes, overexpressing the α subunit decreases acetylcholine sensitivity by less than three-fold. The main difference between the two forms is in the relative potency of DMPP, which is equipotent with acetylcholine on the putative two-α form and 10-fold more potent on the putative three-α form. Interestingly, the rank order of potency of DMPP is also one of the main differences between recombinant and native receptors of this type. Receptors from the soma of autonomic ganglion neurons are most sensitive to cytisine, followed by DMPP and ACh [4], [23], [24], a sequence that is not reproduced by rat subunits expressed in oocytes, and is seen only in a fraction of stably transfected fibroblasts [4], [6]. DMPP is unlikely to be a useful tool in practice, because it is more potent on the low-sensitivity form of the receptor and this reduces the difference in potency in absolute terms. Extrapolation to *EC*_50_s, assuming identical efficacy, suggests that DMPP would have an *EC*_50_ of 30 and 140 µM on the putative three-α and two-α forms, respectively. For α4β2 receptors the difference in potency between the two forms varies widely across agonists (from complete lack of selectivity to 100-fold difference [9]), so α3β4 agonists with better discrimination may yet be found.

### Single-channel properties of the two forms of the α3β4 channel

The putative two-α form of the channel opens to much longer bursts (100 ms or more *vs* 14 ms) with smaller conductance (26 *vs* 39 pS) than the three-α form. Thus, it resembles the predominant type of opening that we described after 1∶1 expression of rat α3β4 receptors in oocytes and in stably-transfected fibroblasts [5], [6] and may account for the slow kinetics of rat α3β4 receptors in voltage-jump relaxations [25]. Channels similar to the putative three-α form, rare in oocytes, are more common, but still a minority, in stably-transfected fibroblasts. This form resembles neuronal nicotinic receptors recorded from rat superior cervical ganglion [5], [26], [27]. Ganglionic receptors are thought to contain α3 and β4 possibly with α5 [28] especially in somatic receptors [24] but see [29] and mediate fast excitatory synaptic events, which have a time course similar to the burst length of the putative three-α form (cf 13.9 ms [30]) and certainly much shorter than that of the two-α form.

Our results differ from those of a previous study [29] that found that oocyte-expressed α3β4 receptors had mostly short bursts, unless expressed with α5. It is difficult to compare the expression conditions, as subunit ratios are not specified and plasmid construction is different (in the ones we employ, the original untranslated regions were removed and replaced by those of β-globin). Conductance comparisons with this and other studies (see [12]) are impossible when the recording solutions contain different concentration of divalent ions (especially calcium, which affects both nicotinic conductance and kinetics [31]).

In nicotinic channels, conductance is controlled by three rings of charges in the pore-lining M2 domain [32] and by residues in an M3–M4 loop amphipathic helix that lines the intracellular fenestrations of the channel [33]. Because α3 and β4 differ at position 20′ in M2 (Glu vs. Lys), a channel containing three α subunits would have an overall negative charge (−1) in this ring and one containing two α subunits would carry an overall positive charge (+1). In the muscle receptor, mutations that produce the same difference in charge in this positions decreased conductance by 20% [32]. These subunits differ also in the intracellular amphipathic helix. At the −4′ position, α3 has a Gln and β4 an Asp and at the 0′ position α3 has a Lys and β4 a Gln residue. These differences would be expected to oppose the effects of the M2 residues on conductance, but it is difficult to estimate by how much (only charge reversals have been tested in the equivalent positions of α4β2 [34]). Our results would suggest that M2 residues have the greater effect.

It is interesting to note the profound difference in kinetics between the two forms of the receptor. The difference in stoichiometry potentially means a different number of binding sites, and it may be that the α3(+)/α3(−) interface can function as an agonist binding site, a hypothesis that warrants further investigation (the Hill slope of the dose-response curve of the putative three-α form is higher, but this could stem from any number of changes in the kinetic mechanism [35].

Our data show that the α3β4 neuronal nicotinic receptor can exist in two forms that differ in stoichiometry. This may be a general phenomenon for heteromeric neuronal nicotinic receptors which can form from the expression of one α and one β subunit. It will be important to assess if native channels can exist in different stoichiometries (or whether this possibility is abolished by the additional subunits native channels may contain) and whether that has physiological or pharmacological consequences.

Note added in print:

During the revision of this manuscript, a report was published showing that, in substantial agreement with our findings, oocyte expression of rat α3β4 neuronal nicotinic receptors gives rise to a mixture of three-α and two-α receptor forms which have differ in their sensitivity to isomers of α-conotoxin AuIB (Grishin AA, Wang CI, Muttenthaler M, Alewood PF, Lewis RJ et al. (2010) α-conotoxin AuIB isomers exhibit distinct inhibitory mechanisms and differential sensitivity to stoichiometry of α3β4 nicotinic acetylcholine receptors. J Biol Chem 285: 22254–22263).

## Materials and Methods

### Preparation of cDNA and cRNA constructs

cDNAs for the human α3 and β4 nAChR subunits (GenBank accession numbers Y08418 and Y08416, respectively) containing the coding sequences and an added Kozak consensus sequence (GCCACC) immediately upstream of the start codon were subcloned into the pcDNA3.1 vector (Invitrogen, Breda, The Netherlands) for expression in human embryonic kidney 293 (HEK293) cells. The 9′ LT mutated subunits (α3^L279T^ and β4^L272T^, where L stands for leucine and T stands for threonine; referred from here on as α3^LT^ and α3^LT^), were created using the QuickChange™ Site-Directed Mutagenesis Kit (Stratagene) and their full-length sequence was verified.

For expression in oocytes, all cDNAs were subcloned into the pSP64GL vector, which contains 5′ and 3′ untranslated regions (UTRs) for the *Xenopus* β-globin. The cDNA/pSP64GL plasmids were linearized immediately downstream of the 3′ UTR, and capped cRNAs were transcribed using the SP6 mMessage mMachine kit (Ambion, Cambridge, UK). The quality of the cRNA molecules was checked by RNA gel-electrophoresis and by comparison with RNA size and concentration markers.

### HEK293 cell culture and transfection

HEK293 cells (ATCC-CRL-1573; American Type Culture Collection) were maintained in a 95% air/5% CO_2_ humidified incubator at 37°C in Dulbecco's modified Eagle's medium (DMEM), supplemented with 0.11 g/L sodium pyruvate, 10% (v/v) heat-inactivated foetal bovine serum, 100 U/ml penicillin G, 100 µg/ml streptomycin sulphate, and 2 mM L-glutamine (all from Invitrogen, Breda, The Netherlands). Cells were passaged every 2–3 days (up to 20 times) and were plated on polylysine-coated coverslips in 35 mm culture dishes, approximately 24 hrs before transfection with a calcium phosphate-DNA co-precipitation method [36]. Cells were transfected with cDNA mixtures for α3β4, α3^LT^β4 or α3β4^LT^. For whole-cell recordings the α3 to β4 cDNA ratio in the mixtures was kept equal (1∶1), whereas for single-channel recordings we used extreme ratios of 1∶9 or 9∶1 of wild-type subunit cDNAs. In all cases, plasmid coding for the marker enhanced green fluorescent protein (eGFP-c1; BD Biosciences, Oxford, UK) was also co-transfected in order to visualise successfully transfected cells. Each culture dish was transfected with 2–3 µg of DNA; this amount was enough for a high rate of transfection efficacy [36]. All recordings were performed 14–48 hrs after transfection, at room temperature.

### *Xenopus* oocyte preparation and cRNA injection

Female *Xenopus laevis* frogs, anaesthetized by immersion in neutralized ethyl m-aminobenzoate solution (tricaine, methanesulphonate salt; 0.2% solution w/v, Sigma), were killed by concussion immediately followed by decapitation and destruction of the brain and spinal cord before removal of the ovarian lobes. This procedure is in accordance with United Kingdom Home Office regulations. The study protocol does not fall under the remit of the UCL Research Ethics Committee (see http://www.ucl.ac.uk/research/images/research-ethics-framework).

Clumps of stage V–VI oocytes were dissected in a sterile modified Barth's solution consisting of (in mM): 88 NaCl, 1 KCl, 0.82 MgCl_2_, 0.77 CaCl_2_, 2.4 NaHCO_3_, 15 Tris-HCl, with 50 U/ml of penicillin and 50 µg/ml of streptomycin (Invitrogen, Breda, The Netherlands) in HPLC water; pH 7.4, adjusted with NaOH. The dissected oocytes were treated with collagenase (type IA, Sigma; for 50–65 minutes at 18°C, 245 collagen digestion units/ml in Barth's solution, 10 to 12 oocytes per ml), then rinsed and stored at 4°C overnight. Treated cells were manually defolliculated the following day prior to cRNA injection. The cRNA mixtures of α3β4, α3^LT^β4 or α3β4^LT^ (in a α3∶β4 ratio of 1∶9 and 9∶1) were injected into oocytes using a Nanoject Automatic Oocyte Injector (Drummond Scientific, Broomall, PA). The total amount of cRNA injected (in 46 nl of RNase-free water, 0.25–10 ng/cell) was determined empirically, with the aim of achieving a maximum ACh-evoked current no greater than 2 µA, in order to contain the magnitude of series resistance errors in our recordings. After injection, the oocytes were incubated for ∼48–60 hrs at 18°C in fresh Barth's solution containing 5% heat-inactivated horse serum (Invitrogen, Breda, The Netherlands) and stored at 4°C. Experiments were carried out at 18°C, 2.5 to 5 days from injection.

### Two-electrode voltage clamp recordings from *Xenopus* oocytes

Oocytes, held in a 0.2 ml bath, were perfused at 4.5 ml/min with modified Ringer solution containing (in mM): 150 NaCl, 2.8 KCl, 10 HEPES, 2 MgCl_2_ and 0.5 µM atropine sulphate; pH 7.2, adjusted with NaOH. The solution was (nominally) calcium-free in order to minimize the activation of calcium-gated chloride channels (endogenous to the oocyte) by calcium entry through nicotinic channels. Oocytes were held at −70 mV using the two-electrode clamp mode of an Axoclamp-2B amplifier (Molecular Devices, CA, USA). Electrodes were pulled from thin-walled Clark borosilicate glass GC150TF (Harvard Instruments) and were back-filled with 3 M KCl (resistance of 0.5–1 MΩ on the current-passing side).

Agonist solutions (freshly prepared from frozen stock aliquots) were applied via the bath perfusion at 5 min intervals for a period sufficient to obtain a stable plateau response (at low concentrations) or the beginning of a sag after a peak (at the higher concentrations). The 5 min intervals were sufficient to ensure reproducible responses. Agonist responses were recorded on a flat bed chart recorder (Kipp & Zonen, Lincoln, UK) for later analysis. A standard concentration of ACh (approximately *EC*_20_) was applied every third response to monitor agonist sensitivity throughout the experiment and allow correction of the data for rundown, as described previously [13]. Experiments were started only after checking that this standard concentration gave reproducible responses. For ACh dose-response curves, a descending dose protocol was used.

For the potency-ratios experiments, low agonist concentrations were tested in each cell in order to obtain partial concentration-response curves (2–3 points for each agonist). The purpose of this was to reduce the impact of desensitization and agonist self-block and to ensure that the slope of all agonist curves was similar when dose-response curves were plotted on double-logarithmic coordinates. Care was also taken to match the size of the responses to different agonists. In all cells, ACh was used as the standard agonist. The maximum agonist concentrations for the 1∶9 and the 9∶1 ratio of α3 to β4were, respectively: 10 nM and 20 nM for epibatidine; 10 µM and 2 µM for lobeline; 25 µM and 5 µM for 1,1-dimethyl-4-phenylpiperazinium iodide (DMPP); 20 µM for nicotine and cytisine; 50 µM for ACh; 0.5 mM and 1 mM for carbachol.

The effect of Zn^2+^ was assessed by co-applying increasing concentrations of Zn^2+^ together with ACh (at its *EC*_20_ concentration, 120 and 50 µM ACh for the 9∶1 and 1∶9 α∶β ratios, respectively). Before each application, oocytes were preincubated for 30 s with Zn^2+^ alone. ACh responses in the presence of Zn^2+^ were normalised to the response from ACh alone, at the same concentration.

### Whole-cell recordings from HEK293 cells

Whole-cell recordings from transfected HEK293 cells were performed with an Axopatch 200B amplifier (Molecular Devices, CA, USA) at a holding potential of −30 mV. Cells were superfused with extracellular solution containing (in mM): 120 NaCl, 3 KCl, 3 HEPES, 2 CaCl_2_, 2 MgCl_2_, 25 D-glucose, pH 7.4 (adjusted with Tris base). Electrodes (2–4 MΩ final resistance) were pulled from thin-walled borosilicate glass GC150TF (Harvard Instruments) and backfilled with a solution containing (mM): 110 Tris-phosphate dibasic, 28 Tris-base, 11 EGTA, 2 MgCl_2_, 0.1 CaCl_2_, 4 Na-ATP, pH 7.3 (adjusted with Tris base). This K^+^-free intracellular solution was adopted in order to minimize the receptor rundown during recording [37]. Series resistance (between 4–10 MΩ) was routinely compensated to 80–95%. Agonist solution (freshly prepared from frozen ACh stock aliquots), was applied through a modified U-tube system with solution exchange times of the order of 2–6 ms, measured by recording the open tip potential change to the application of a 50% diluted extracellular solution before the experiment. Different ACh concentrations were applied at 2 min intervals in a random order and a standard concentration of ACh (∼*EC*_20_) was applied every third response in order to allow us to monitor for rundown.

Whole-cell records were filtered at 0.5 kHz and digitized at 2 kHz to a computer with a Digidata 1322A interface and Clampex software (both from Molecular Devices, CA, USA).

### Single-channel recordings

Recordings from HEK293 cells were performed in the outside-out configuration at −100 mV. The extracellular solution contained (in mM): 150 NaCl, 1.1 KCl, 2 MgCl_2_, 1 CaCl_2_, 10 HEPES, pH 7.2 (adjusted with NaOH). Electrodes were pulled from thick-walled Clark borosilicate glass GC150F (Harvard Instruments), coated near the tip with Sylgard 184® (Dow Corning, UK) to improve their dielectric properties and fire-polished prior to use, to a final resistance of 8–12 MΩ. Electrodes were filled with high potassium intracellular solution to minimise the contribution of potassium channels at the holding potential used for recording (−100 mV). The solution contained (in mM): 107.1 KCl, 1 CaCl_2_, 1 MgCl_2_, 10 HEPES, 11 EGTA, 20 TEACl, 2 MgATP (pH was adjusted to 7.3 with KOH and osmolarity was corrected to 320 mOsm with sucrose when needed).

Single-channel currents, elicited by the application of 5 µM ACh, were recorded with an Axopatch 200B amplifier, prefiltered at 10 kHz (using the 4-pole Bessel filter of the amplifier) and stored on a DAT tape (Bio-Logic Science Instruments, Claix, France). For off-line analysis, recordings were played back from the tape, filtered at 0.5–2 kHz with a 8-pole Bessel filter, and digitized to a computer with a sampling rate of 30 kHz, using a Digidata 1322A and Clampex.

### Data Analysis: whole cell responses

ACh dose-response curves obtained either from HEK293 cells or oocytes, and also Zn^2+^ dose-response curves from oocytes were fitted with the Hill equation:
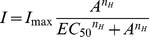
(1)where *I* is the current response, measured at its peak, *I*_max_ is the maximum response, A is the agonist concentration, *EC*_50_ is the agonist concentrations for 50% maximum response and *n*_H_ is the Hill coefficient. The program CVFIT, courtesy of D. Colquhoun and I. Vais, (available at http://www.ucl.ac.uk/Pharmacology/dc.html) was used for least square fitting. Data for each cell or oocyte were fitted separately to give the values for *I*_max_, *EC*_50_, and *n*_H_ in the Tables (shown as mean ± standard error of the mean).

In order to estimate the shift in the dose-response curve caused by the 9′ LT mutation, individual curves were normalised each to its fitted maximum and pooled, to give one normalised curve for each receptor combination. These sets were then fitted again simultaneously with the Hill equation, with the constraint of having equal Hill slopes (“parallel” fits). From the parallel fits, the program could estimate the distance between the wild-type and mutant curves (i.e. the dose ratios, see Table 1) and the 2.01-unit likelihood intervals for the dose ratios, which are equivalent to a confidence interval of ±2 standard deviations for a Gaussian variable [38].

For the potency ratios experiments, all partial dose-response curves obtained from each oocyte were fitted simultaneously with equal weighting, using CVFIT, with power functions constrained to be parallel. This is equivalent to fitting the dose-response curves with a Hill equation, with the constraint of having a *I*_max_ which is much higher than the responses analysed (see equation 1; as Figure 2 shows, the responses to the concentrations of the standard agonist used in these experiments, ACh, were less than 10% of *I*_max_); at low concentrations, the slope of this log-log plot (Figure 4B) tends to the value of the Hill slope. For each cell, dose ratios for each agonist were estimated relative to ACh (equal to dose ratio of 1). Potency ratios for each agonist are the reciprocals of the dose ratios (i.e. equivalent to saying that each agonist is x times more potent than ACh). Errors and confidence intervals for potency ratios were calculated using Fieller's theorem [39]. In order to confirm that the curves were indeed parallel, all data were also fitted without the constraint of parallelism to obtain the slope for each individual agonist (Table 3).

The dose-response curve to zinc was fitted to the sum of two Hill equations (parameterised as logistic equation by the fitting program Prism 5.01, GraphPad Software, San Diego, USA):
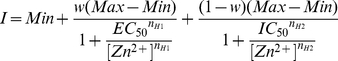
(2)where *I* is the ACh response, measured at its peak and normalized to the value in the absence of Zn^2+^, *w* is a fitted coefficient between 0 and 1 that gives the weight of the first component, *Max* and *Min* are fitted parameters that express the maximum enhancing or inhibiting effect of Zn^2+^ (as a fraction of the control response), *EC*_50_ and *IC*_50_ are the Zn^2+^ concentrations for 50% maximum enhancement or inhibition, respectively, *n*_H1_ and *n*_H2_ are the Hill coefficients of the two components and *x* is log_10_ [Zn^2+^]. Data from each experiment were fitted separately and the resulting *EC*_50_ values were used to obtain the mean *EC*_50_ and the standard error of the mean reported in the text. For the experiments in Figure 5B, the fitted values of *w*, *Max* and *Min* were 0.31, 1.78 and −0.83, respectively.

### Data analysis: single channels

The recordings chosen for single-channel analysis were idealized by time-course fitting using the program SCAN. After choosing and imposing the appropriate resolution (40–200 µs), fitted amplitude diagrams were created and fitted with the appropriate number of Gaussian curves using EKDIST (both programs are available at http://www.ucl.ac.uk/Pharmacology/dc.html). More than 100 openings were used from each experiment and these openings were included only if they were longer than twice the rise time of the filter and therefore had well-defined amplitudes. Current amplitudes were converted into chord conductances, assuming a reversal potential of 0 mV (with no correction for junction potential). For patches with both high- and low-conductance openings, burst duration was analysed separately for the two channels.
